# Current Knowledge and Utilization of Medicinal Plants and Fungi in Northeastern Croatia

**DOI:** 10.3390/plants15020325

**Published:** 2026-01-21

**Authors:** Ljiljana Krstin, Zorana Katanić, Ivana Turk, Ivana Gajski, Tanja Žuna Pfeiffer

**Affiliations:** Department of Biology, Josip Juraj Strossmayer University of Osijek, 31000 Osijek, Croatia; lkrstin@biologija.unios.hr (L.K.); zkatanic@biologija.unios.hr (Z.K.); turk.ivana111@gmail.com (I.T.); ivana.gajski7@gmail.com (I.G.)

**Keywords:** ethnobotany, traditional knowledge, wild plants, cultivated plants, medicinal plants

## Abstract

Knowledge related to the use of plants and mushrooms in the Baranja region of Croatia was documented through semi-structured interviews of 105 informants in 12 villages. We found 117 plant species and 7 mushrooms with medicinal uses. Rosaceae, Lamiaceae, and Asteraceae were the families with the most species, while *Sambucus nigra*, *Chamomilla recutita*, and *Taraxacum officinale* were the most frequently mentioned species. Leaves, fruits, and flowers were the most commonly used plant parts, predominantly prepared as infusions, syrups, and tinctures. Plants were mainly used to treat digestive and respiratory ailments, with the highest informant consensus recorded for ear, eye, and respiratory disorders. The results emphasize the persistence of rich ethnobotanical knowledge in the study area and highlight the importance of preserving this cultural and biological heritage for future generations.

## 1. Introduction

Since ancient times, plants have been essential to human life. They provide nutrients and construction materials, as well as natural medicines whose therapeutic properties are used to prevent and treat numerous diseases [1,2]. Early medicinal plant use involved simple herbal preparations, and knowledge of the therapeutic properties and application methods of specific plants for treating various ailments was transmitted orally across generations. Over time, this traditional knowledge has been systematically recorded and preserved [3]. However, with the advancement of modern medicine and technology, the traditional use and knowledge of medicinal plants have gradually declined. Numerous traditional healing methods have been marginalized and forgotten over time, resulting in decreased everyday use of medicinal plants [4,5].

Recently, an increasing number of people have begun to rediscover the value of nature, turning to medicinal plants as a more natural and healthier alternative. Natural remedies and traditional knowledge about plants are once again gaining importance, not only for health preservation but also as part of a sustainable lifestyle [6]. Today, many plants are essential sources of medicinal compounds. Modern pharmaceutical research often relies on knowledge derived from ethnomedicinal practices in the development of new, effective therapies with fewer side effects [7]. Such research enables the isolation of bioactive plant compounds that can be applied in the treatment of various diseases [8,9]. There is a growing interest in integrating traditional knowledge with modern science to develop safer and more natural therapeutic solutions [10].

In addition to plants, mushrooms are also an important source of food and natural medicinal substances. Many mushroom species are known for their antimicrobial, anti-inflammatory, antitumor, and antidiabetic properties. Their bioactive compounds are used in the prevention and treatment of numerous chronic diseases. Traditional knowledge about the medicinal use of mushrooms from various cultures is now becoming the subject of modern scientific research, opening up possibilities for the development of new functional products and natural medicines [11,12]. Therefore, the preservation and study of indigenous knowledge of mushrooms are as valuable as the preservation and study of medicinal plants.

The increasingly pronounced changes in climate further underscore the importance of preserving knowledge about medicinal plants. Climate change has a significant negative impact on plant life and overall biodiversity. Medicinal plants, which often grow in specific habitats, are particularly vulnerable to the risks arising from temperature shifts, changes in precipitation patterns, habitat degradation, and the spread of invasive species. Research indicates that climate change may drastically reduce the habitats of medicinal plants in some regions if appropriate conservation measures are not implemented [13]. Furthermore, climate change can impact the pharmacological properties of plants by altering the concentration and composition of bioactive compounds that determine their medicinal value. This further highlights the importance of traditional knowledge for the identification, harvesting, and use of these plants [14]. Knowledge of plant species, especially medicinal ones, is essential for developing protection strategies for natural habitats. Therefore, collaboration among local communities, scientists, and policymakers is crucial for protecting plant species and preserving biodiversity [15].

Thanks to its geographical location, climatic diversity, and well-preserved natural habitats, Croatia stands out as a country of exceptionally rich biodiversity. A large number of indigenous and wild plant species grow in its territory, including many medicinal plants traditionally used in folk medicine [16,17]. Because of this natural wealth, ethnobotanical studies have been conducted in certain areas of Croatia. For example, research has been conducted in the north-eastern part of Croatia [18], the continental northwestern region [19,20], the mountainous interior of Dalmatia, and in the areas of Knin [21] and Lika [22]. Although previous research has made a significant contribution to the preservation of valuable ethnobotanical knowledge, a considerable number of Croatian regions remain insufficiently explored. Many communities, especially in rural areas, still preserve rich knowledge about medicinal plants and their traditional uses, which has not yet been systematically recorded or analyzed. For this reason, the present study focused on Baranja, a unique region in the far east of Croatia that is characterized by a rich natural and cultural heritage.

The objectives of the study were (1) to identify plant species and mushrooms traditionally used among the local population, (2) to identify plant species and mushrooms used for medicinal purposes, (3) to distinguish plants and mushrooms with a maximum frequency of citation (FC), (4) to determine consensus levels among the informants based on the informant consensus factor (ICF), and (5) to discuss the significance of these species for the local community.

## 2. Results

**Figure 1:**
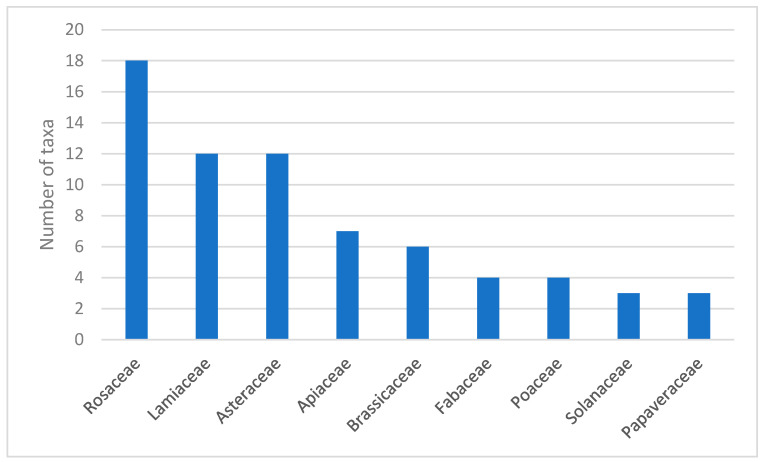
Plant families with the highest number of taxa mentioned by informants during an ethnobotanical study in the Baranja region (Croatia).

A total of 117 species were recorded, which are listed in alphabetical order in Table S1. These plants are classified into 45 families and 93 genera. The family Rosaceae had the widest diversity with 18 taxa recorded, followed by Lamiaceae (12 taxa) and Asteraceae (12 taxa) (Figure 1). Eight families were represented by only one taxon.

Among the taxa, approximately 61% were herbaceous plants, 22% were trees, and almost 15% were shrubs. There were also two climbers and one hemiparasite. The species with the highest frequency of citation (FC) and use report (UR) values were *Sambucus nigra* L. (FC = 62; UR = 103), *Chamomilla recutita* (L.) Rauschert (UR = 53, UR = 87), and *Taraxacum officinale* F. H. Wigg. (FC = 43, UR = 65) (Table S1).

The frequency of citation (RFC) ranged from 0.01 to 0.59. *S. nigra* had the highest RFC value (0.59), followed by *C. recutita* (0.51), *T. officinale* (0.41), *Tilia cordata* Mill. (0.25), *Juglans regia* L. (0.23), and *Urtica dioica* L. (0.21). The minimum RFC value of 0.01 was recorded for 37 species (Table S1).

The informants cultivate 64 plants and gather 53 wild plants from nature (Table S1). The plants are mostly collected from yards, gardens, fields, forests, and field or forest paths. The most utilized plant parts are leaves (41 taxa), followed by fruits (36 taxa), flowers (34 taxa), herbs (14 taxa), roots (11 taxa), and seeds (11 taxa). Some of the informants also reported using bulbs (*Allium sativum* L.), onion peel (*A. cepa* L.), petioles (*Prunus avium* L.), bark (*Salix caprea* L., *Quercus* sp.), and tubers (*Solanum tuberosum* L.). Infusion is the most common method of preparation (64 taxa), followed by fresh use (42 taxa), syrup (24 taxa), jam (19 taxa), liqueur (16 taxa), spice (15 taxa), tincture (14 taxa), compress (13 taxa), dry and as juice (8 taxa), as well as cake and oil (7 taxa). Other preparation methods mentioned by the informants were decoctions (6 taxa), macerates, creams, compotes (5 taxa), honeys, balms, ointments (4 taxa), schnapps, salads (3 taxa), and wines (2 taxa).

The majority of the plants, including *A. sativum*, *Cornus mas* L., *Morus nigra* L., *T. officinale*, and *Melissa officinalis* L., were used for both medicinal and food purposes (64 species), while 53 species were only used for medicinal purposes.

The informants reported using various plants for food preparation, making different food products such as soups (*U. dioica*), vegetable stews (*Anethum graveolens* L., *Capsicum annuum* L.), pies (*Prunus domestica* L.), cakes (*J. regia*, *Corylus avellana* L.), salads (*Plantago major* L., *T. officinale*), smoothies (*Petroselinum crispum* Mill. A. W. Hill), fritters (*S. nigra*), wines (*Vitis vinifera* L., *Rubus caesius* L.), schnapps (*P. domestica*), juices (*S. nigra*), and honey (*Robinia pseudoacacia* L., *T. cordata*). Additionally, the informants reported preparing various winter food products from certain plants that they use very often during the winter months, such as jam (*Crataegus monogyna* Jackq., *Rosa canina* L.), compote (*Cydonia oblonga* Mill.), dried foods (*C. avellana*, *J. regia*), pickles (*Beta vulgaris* var. *conditiva* L.), and vinegar (*Malus sylvestris* Mill.).

Additionally, the informants prepared a variety of non-alcoholic beverages (*R. caesius*, *S. nigra*, and *Cichorium intybus* L.) and alcoholic drinks (*V. vinifera*, *J. regia*, *Mentha* × *piperita* L., *P. domestica*, and *P. cerasus* L.) from plants. They also used them for livestock feed (*Quercus* sp., *Cucurbita pepo* L., *U. dioica*), plant supplements (*U. dioica*), and insecticides against pests (*J. regia*, *Artemisia vulgaris* L., *U. dioica*). Moreover, the plants were utilized for decorations (*Lavandula angustifolia* Mill, *S. caprea*), as space fresheners (*L. angustifolia*), as mosquito and moth repellents (*M. × piperita*, *L. angustifolia*), as thermal seed bags (*P. cerasus*), and for dyeing Easter eggs (*A. cepa*).

A total of 1129 use reports for medicinal purposes were mentioned. These documented plants were classified into 14 ICPC-2 disease categories (Table 1).

Some plants were reported to be effective in treating only one disease and were therefore placed in a specific disease category. In contrast, other plants were classified into several categories and were used to treat more than one disease. For instance, *C. recutita* and *U. dioica* were recorded in 10 categories; *S. nigra*, *T. officinale*, and *Rosmarinus officinalis* L. were recorded in 9; and *H. perforatum*, *J. regia*, and *R. canina* were recorded in 9 categories. Most categories contained plant species mentioned only once by informants, resulting in a single-mentioned item index ranging from 0.26 to 0.72.

The number of use reports per usage category ranged from 14 to 227. The most frequently treated diseases with the medicinal plants in the study area are diseases of the digestive system (constipation, diarrhea, stomach pains, heartburn, and stomach ache) with a UR of 227; respiratory system (cold, sore throat, inhalation, laryngitis, and cough) with a UR of 176; blood, blood-forming organs, and immune system (blood strengthening, anemia, and improving blood count) with a UR of 164; cardiovascular system (cholesterol, hypertension, varicose veins, hemorrhoids, and blood circulation) with a UR of 124; and skin (rash, wound, warts, burn, and acne) with a UR of 119. The lowest UR value of 14 was related to earaches (Table 1).

To treat digestive system diseases, the informants use 55 taxa, with *S. nigra* (UR = 18), *Mentha* × *piperita* (UR = 18), and *C. recutita* (UR = 15) being the most frequently mentioned. To treat cardiovascular system diseases, the informants use 46 species, with *C. monogyna* (UR = 12), *J. regia* (UR = 8), and *M. pumila* (UR = 8) being the most prominent. A total of 39 plant species are used to treat blood and immune system diseases, with *S. nigra* (UR = 30), *U. dioica* (UR = 13), and *C. recutita* (UR = 11) having the highest UR values, while 29 plants are used to treat urological diseases; the most commonly mentioned plants were *T. cordata* (UR = 15), *T. officinale* (UR = 13), and *U. dioica* (UR = 7). Additionally, 29 plant species are used to treat skin diseases, with *C. officinalis* (UR = 20), *C. recutita* (UR = 18), and *H. perforatum* (UR = 17) being the most frequently mentioned. Moreover, 29 plant species are used to treat respiratory diseases, among which, the most frequently mentioned were *S. nigra* (UR = 42), *T. cordata* (UR = 22), and *C. recutita* (UR = 15). In the category of endocrine, metabolic, and nutritional diseases, 19 plant species were recorded, with the most common being *T. officinale* (UR = 8), *J. regia* (UR = 4), and *C. pepo* (UR = 3), while 18 species were listed in the category of musculoskeletal diseases, with the most popular being *Brassica oleracea* ssp. *capitata* (L.) Duchesne (UR = 9), *Symphytum officinale* L. (UR = 9), *P. crispum* (UR = 4), and *H. perforatum* (UR = 3). In the other categories, fewer than 15 plants were recorded, and *Sempervivum tectorum* L. was the only plant recorded for the treatment of ear diseases (Table 1).

Since some medicinal species are used to treat diseases across multiple categories, the informant consensus factor was calculated across these disease categories. The ICF values ranged from 0.39 to 1. The highest ICF value of 1 was recorded for the treatment of ear disorders, followed by eye disorders (0.95); respiratory system disorders (0.84); blood, blood-forming organ, and immune mechanism disorders (0.77); and skin diseases (0.76). The lowest ICF value (0.39) was recorded for general and unspecified disorders (Table 1).

A total of seven mushroom species belonging to six different families were recorded. The most frequently mentioned species was *Laetiporus sulphureus* (Bull.) Murrill, commonly known as “*Chicken of the Woods*”. The informants described various methods of mushroom preparation, including making soups, sautéing, frying, and breading. Mushrooms were also prepared in combination with eggs or pasta and were often added to stews and goulashes to enhance the nutritional value and flavor of the dishes (Table 2).

## 3. Discussion

The results of an ethnobotanical study in settlements of the Baranja region showed that 117 plant species have ethnomedicinal applications among the local population. These results are consistent with those of other studies conducted in different parts of Croatia, which showed that rural populations use plants for various purposes [19,22]. In this study, women comprised the majority of the informants (approximately 65%). Previous ethnobotanical research also reported that women are often involved in the transmission of knowledge about medicinal plants [23]. However, due to the uneven gender distribution in the sample, it is not possible to conclude that women play a particularly important role in preserving traditional knowledge in the study area. Furthermore, only about 10% of the informants were in the 35–45 age group, suggesting a lower representation of traditional knowledge among younger individuals, likely related to lifestyle modernization, reduced interest in traditional practices, and the dominant influence of modern medicine and pharmaceutical solutions [24]. However, the uneven age distribution of the informants limits the ability to draw reliable conclusions about age-related differences in knowledge. Similar patterns (lower representation of younger informants) were also found in other ethnobotanical studies [23,24].

Plants uses in the Baranja have many similar elements with other areas but also have some unique features (Table S1), indicating that some plants have multiple beneficial effects and their uses mainly depend on traditional knowledge as well as on availability. The recorded species demonstrate a high diversity, encompassing 45 families and 93 genera. The dominance of certain families is consistent with findings from other ethnobotanical studies in Europe and the Mediterranean region. Among the most represented families, Rosaceae stands out with 18 taxa, reflecting its importance in traditional medicine and nutrition. This family has a cosmopolitan distribution, and species are often used due to their availability and versatile applications [25]. For example, in Italy, species such as *Prunus dulcis* (Mill.) D. A. Webb, *Rosa canina*, and *Rubus fruticosus* L. are regularly used for various medicinal purposes [26], while in Spain, *Rosa pouzinii* Tratt. and *Rubus ulmifolius* Schott are consumed raw as snacks [27]. The families Lamiaceae and Asteraceae, with 12 taxa, were cited by the informants for their aromatic and medicinal properties. Their importance has been recorded in southern, central, and eastern Europe [28], where Lamiaceae ranks first in significance, followed by Asteraceae [26]. Genera such as *Mentha*, *Origanum*, and *Thymus* are particularly prominent [29]. Apiaceae (7 taxa) and Brassicaceae (6 taxa), although somewhat less represented in this study, play an important role, particularly in culinary use and digestive support. Apiaceae are widely used in both Mediterranean and North African cuisines, especially in Morocco, Spain, Turkey, and Armenia, for seasoning and improving digestion [30]. Brassicaceae are also used mainly for food and in local healthcare practices in Sicily [31] and Pakistan [32].

RFC, together with the UR and ICF indices, was used to compare the cultural importance of plant species and the level of consensus among the informants [33]. Although these quantitative indices have certain methodological limitations [34], they were selected for their wide applicability and methodological simplicity in ethnobotanical research [35,36]. The species *S. nigra*, *C. recutita*, *T. officinale*, *T. cordata*, *J. regia*, and *U. dioica* were among the most frequently mentioned plants. The high RFC values for these species correspond to their ecological abundance, as they are widely distributed and abundant in the Baranja region (personal observation). Additionally, the high RFC values for these species indicate their greater cultural importance within the local community [37]. Furthermore, some of the most frequently cited plant species, such as *T. officinale*, represent common, widely distributed, and ecologically abundant taxa. Their abundant availability in the environment likely contributes to their frequent use [38]. *Taraxacum officinale* is frequently used for medicinal purposes and as a food in the Baranja region. The informants (43 of them) reported using this species for respiratory problems (e.g., cough and bronchitis), gastrointestinal issues, improving digestion, blood purification, cardiovascular health, liver health, blood sugar regulation, and diabetes. It was also applied for detoxification, as a diuretic, and to treat urinary tract infections. These applications could be attributed to its therapeutic properties, including diuretic, hepatoprotective, antidiabetic and anticolitics effects. Also, *T. officinale* exhibits antioxidant, anti-inflammatory, antimicrobial and immunoprotective activities, which could explain its use in the treatment of respiratory and urinary infections and gastrointestinal disorders [39,40]. Moreover, 13 informants incorporate *T. officinale* into their diet by consuming it fresh in salads or in the form of infusions, syrups, decoctions, and honey. Due to its widespread recognition, this plant is widely used for therapeutic and other purposes in many countries [41]. In China, it is traditionally used for detoxification and the treatment of hepatitis and respiratory inflammation. In Turkey, it is known to be an effective laxative, a diuretic, and a natural remedy for diabetes, while in Mexico, it is most commonly used for blood sugar regulation and as an antimicrobial agent. In Germany, it is used to treat gout, liver problems, and digestive disorders, while indigenous communities in North America use this plant to alleviate kidney diseases, improve digestion, and treat skin problems and rheumatism [42].

In total, 59% of the informants use *S. nigra* for medicinal purposes and as food. Its flowers and fruits are processed into juices, infusions, jams, syrups, fritters, and liqueurs and used to treat of various ailments such as colds, flu, a weakened immune system, anemia, and gastrointestinal issues. The use of *S. nigra* for the treatment of respiratory infections may be partly attributed to its antiviral activity and immunomodulatory effects. Schön et al. [43] demonstrated that an anthocyanin-enriched fruit extract of *S. nigra* significantly reduces viral infectivity and affects the secretion of cytokines, immune system molecules that regulate inflammatory responses. Furthermore, Stich et al. [44] demonstrated that polysaccharides isolated from *S. nigra* extract enhance T-cell–mediated immune responses, further supporting its potential role in promoting immune function. *Sambucus nigra* is also used in both traditional and modern phytotherapy in other parts of Croatia [20,22]. Worldwide, it is used to promote sweating and relieve cold symptoms [45] and to treat conjunctivitis, constipation, diabetes, diarrhea, dry skin, headaches, and rheumatism [46]. In France and Belgium, it serves as a natural diuretic [47], while in Germany and the United States, it is effective for the treatment of flu and colds in the form of teas, film-coated tablets, and liquid extracts [46]. *Sambucus nigra* juice may help regulate body weight and improve metabolic health. Its consumption positively affects gut microbiota, enhances glucose metabolism, and promotes more efficient fat breakdown [48]. Generally, the positive effects of *S. nigra* are attributed to its high content of anthocyanins, bioactive compounds with proven anti-inflammatory, antidiabetic, and antimicrobial properties [49].

*Chamomilla recutita* is one of the most well-known medicinal plants; it is widely distributed and used by many cultures [50]. In the Baranja region, *C. recutita* in the form of infusions, compresses, inhalations, creams, ointments, balms, and decoctions is most commonly used for the treatment of eye infections, colds, gastrointestinal problems, skin problems (e.g., burns, acne, and rashes), insomnia, anxiety, stomach pain, headaches, urinary tract infections, insect bites, as well as for immune support. Traditional uses can be partly attributed to the rich chemical composition of *C. recutita*, which includes flavonoids, coumarins, volatile oils, terpenes, organic acids, and polysaccharides. These constituents may exhibit anti-infective, neuroprotective, and antidepressant effects, providing pharmacological support for some uses, particularly those related to infections, gastrointestinal disorders, and mild nervous system conditions [51]. However, *C. recutita* compresses may pose a health risk due to potential allergic reactions and microbiological contamination. Due to the lack of scientific evidence, further research is needed on the use of *C. recutita* to treat eye infections [52]. Worldwide, it is used to treat infections and neuropsychiatric, respiratory, gastrointestinal, and liver disorders, probably due to its valuable medicinal properties [50,53]. It has anti-inflammatory and antioxidant effects, can be used to treat cancer and ulcers, and protects the skin while preventing its aging [54]. In Germany, it is considered a universal remedy [55], and due to its gentle yet effective action, it is widely used on children as well [56,57].

*Tilia cordata* was also mentioned by a high number of informants (26), who used this plant to treat gastrointestinal problems, colds, coughs, and headaches; to strengthen the immune system; for detoxification; and as a food. Studies have shown that infusion of *Tilia* sp. contains high levels of phenolic compounds with strong antioxidant and free radical scavenging activity, as well as moderate antimicrobial effects in vitro. These properties may partly explain its traditional use in the treatment of colds and respiratory ailments [58]. Furthermore, procyanidins isolated from *T. cordata* flowers can modulate the activity of human neutrophils by reducing oxidative stress and inflammatory responses in *vitro*. These anti-inflammatory properties may partially support the use of flowers in inflammatory and respiratory conditions [59]. Generally, *Tilia* spp. are widely distributed in Europe, and their uses for various purposes were noted in Central Macedonia, Greece (e.g., respiratory diseases and intestinal pain) [60], Hungarian communities in Slovenia [61], Serbia [62], the Czech Republic [63], as well as in other parts of Croatia [19,20].

The informants use *U. dioica* for different medicinal purposes, including anemia, dermatological problems, rheumatism, immune system strengthening, urinary tract problems, and gastrointestinal problems. This use is consistent with its rich chemical composition, including vitamins, minerals, flavonoids, and chlorophyll, which contribute to its anti-inflammatory, diuretic, antianemic, immunostimulant, and hypoglycemic effects [64]. In addition, studies have shown that polyphenols isolated from the leaves and flowers showed strong antioxidant effects, reduced oxidative stress, and inhibited pro-inflammatory cytokines in human skin cells, without cytotoxic effects. These findings may support the traditional use of *U. dioica* for skin disorders [65]. Its medicinal value is recognized in traditional medicine in Serbia [66] and Bosnia and Herzegovina [67].

Due to its diverse phytochemicals and fat-soluble bioactive, nutrient, and non-nutrient antioxidants, *J. regia* has many medicinal properties, antioxidant, anti-inflammatory, antimicrobial, antidiabetic, neuroprotective, hepatoprotective [68], and contains valuable food additives [69]. Thus, it is not surprising that a high number of informants (24) use this species as food and for medicinal purposes, such as improving thyroid function; treating diabetes, anemia, and gastrointestinal problems; and improving prostate health. This species is widely used traditionally in neighboring Serbia [46], Bosnia and Herzegovina [67], and Italy [70]. In addition, like many medicinal plants, its extracts exhibit allelopathic effects on various species [71], indicating its potential for use in the development of bioherbicides for eco-friendly agriculture [72].

Plants other than *J. regia* can also have toxic effects (Table S1). For example, in traditional medicine, *Chelidonium majus* L. is commonly used externally to remove warts and treat other dermatological conditions [73]. The informants in this study reported using *C. majus* to treat corns. This species has been associated with a variety of pharmacological activities, including antibacterial, antifungal, anti-inflammatory, antiviral, and antitumor effects. However, the alkaloids found in *C. majus*, either alone or in combination, can be toxic, especially to the liver, affecting its function through different mechanisms [74]. Furthermore, *Petasites hybridus* (L.) P. Gaertn., B. Mey. et Schreb, which the informants in this study reported using fresh as an analgesic, has been utilized in traditional European medicine for the treatment of various ailments [75]. Many of this plant’s active compounds have medicinal properties, but it also contains pyrrolizidine alkaloids, which exhibit acute toxicity when consumed in high amounts and chronic toxicity upon long-term consumption, which can lead to liver damage, liver carcinoma, hemangioendothelial sarcoma, and tumors in the lungs, pancreas, and intestines [76]. To avoid injury or life-threatening conditions, plants should be used with caution and with knowledge of appropriate application doses [77].

The results showed that growing plants in the gardens, yards, and fields represents an important source of 64 species for the local population. Worldwide, home gardens are primarily used for food cultivation, although many of the plants grown could also be used for medicinal purposes [78,79]. This practice was also noted in a previous study in Croatia [19]. Among various cultivated taxa, the most common were *B. oleracea* L. ssp. *capitata*, *C. officinalis*, *Daucus carota* L., and *P. cerasus*. Most of the cultivated plants are native, but some non-native species also occur. One such plant is *J. regia*, which originated from Central Asia, but is now cultivated worldwide [80] and is also grown in the gardens of many of the informants. *Laurus nobilis* L., *L. angustifolia*, and *R. officinalis* are not native to the Baranja region either, but their uses indicate good adaptation to continental conditions and their acceptance by the local population. *Aloe vera* L., which originated in the Arabian Peninsula and is cultivated worldwide [81], as well as *Nigella sativa* L., indigenous to the Mediterranean region [82], have been cultivated in Baranja and are used for skin care and digestive problems, respectively. This could be associated with their well-known pharmacological properties and the growing popularity of using these species for medicinal purposes [83,84]. These results suggest that the current knowledge and plant use in Northeastern Croatia represent a blend of ethnobotanical heritage and modern knowledge of plants.

The most commonly used plant parts among the informants are leaves, fruits, and flowers. Similar results have been reported in other ethnobotanical studies [85]. Leaves are easily accessible and simple to collect, and their use does not require damaging the plant. Besides these practical advantages, they contain significant amounts of phytochemicals, essential oils, and secondary metabolites, which explains their frequent application in traditional medicine [86]. Fruits and flowers, although somewhat less represented, also stand out for their nutritional and medicinal properties.

The most common method of preparing medicinal plants among the informants is preparing infusions, which is consistent with previous ethnobotanical studies [35,62,87,88]. The use of infusion as a common form of application can be attributed to its simplicity in preparation, the culturally established practice of consumption, and its satisfactory effectiveness in extracting biologically active compounds from plant material.

The informants also use plants to prepare various alcoholic and non-alcoholic beverages, different types of dishes, and winter supplies such as compotes, jams, and dried fruits, which further emphasizes their importance in daily life and the preservation of food security, especially in this predominantly agricultural area where plant cultivation is closely linked to local nutrition and household self-sufficiency [89]. Additionally, plants are used for crop protection, as livestock feed, for decorative purposes, and to freshen spaces, reflecting the multifunctional role of plants in rural life.

The most common uses of medicinal plants were to alleviate digestive and respiratory ailments. These two categories of illnesses were consistently highlighted as the most prevalent in numerous other ethnobotanical studies [90,91]. Given the tradition of using medicinal plants, the availability of plant resources, and the trust in their effectiveness, the local population often uses plants for self-treatment of common, but not necessarily serious, health issues, such as respiratory and digestive problems. This form of self-care is based on knowledge passed down through generations, which still plays an important role in everyday healthcare practices, especially among elderly people. Similar results have been reported in other studies conducted in Iran [23], Ethiopia [24], and Turkey [36], in which the most frequently reported uses of medicinal plants were alleviating digestive and respiratory ailments. A total of 55 plant species are used to treat digestive disorders, with *S. nigra*, *M. × piperita*, and *C. recutita* being the most frequently cited. The high number of taxa recorded for digestive disorders may be explained by a combination of cultural and ecological parameters. Digestive ailments play a central role in everyday health and are traditionally treated with a wide variety of plant-based remedies, leading to high taxonomic redundancy. Moreover, many plants used for digestive purposes are ecologically abundant and easily accessible, increasing their likelihood of citation.

The high number of taxa used for digestive disorders is consistent with other ethnobotanical studies in the Central Balkans [66] and Turkey [36]. On the other hand, the ear category only included a single species, which could artificially inflate the ICF value. Other studies have also reported high ICF values for categories with only one species [92].

In addition to plants, the informants recognize and use wild mushrooms in their diet. Although they do not constitute a dominant part of food resources, mushrooms hold a significant place in the informants’ traditional diet. A total of seven mushroom species belonging to six different families were recorded, with the most frequently mentioned species being *L. sulphureus*. This mushroom stands out not only for its availability but also for its culinary value thanks to its texture and taste reminiscent of poultry. This species has also been highlighted in previous studies as one of the most commonly consumed mushrooms in rural areas of Central and Southeastern Europe [93,94]. The informants reported various methods of mushroom preparation, and their use in diverse dishes suggests that the respondents possess knowledge of mushrooms’ nutritional properties, including their ability to enhance the flavor and nutritional value of meals. Mushrooms are a valuable source of protein, fiber, vitamins, and minerals, and their regular consumption can help maintain health and prevent numerous chronic diseases [95]. Similar findings have been recorded in other parts of Croatia and the wider region. For example, a study conducted in north-western Slavonia documented 28 fungal taxa used for food, while 17 were recorded in Valpovo and Đurđevac [19,20]. In Lika, the lesser-known parasitic species *Taphrina pruni* (Fuckel) Tul. is used [22], and in Bosnia and Herzegovina (municipality of Vitez) and Dalmatian Zagora, numerous edible mushroom species that form part of the local populations daily diet have been recorded [96,97]. These data confirm the importance of mushrooms in the dietary and cultural heritage of rural communities and emphasize the need to preserve this valuable knowledge about their use and application in everyday life.

However, the results indicate that the study could be affected by several potential limitations. The selection of informants and the study area may have influenced the documented plant diversity, as knowledge distribution within the community and the region is not homogeneous. The informants’ sources of knowledge were not systematically assessed, which may have influenced the reporting and frequency of specific plant uses. Consequently, the documented plant uses may reflect a mixture of traditional knowledge and knowledge acquired from other sources, books, mass media, and/or popularized scientific information. Furthermore, recall bias and cultural salience may have affected the frequency of cited species, particularly those with high RFC values, and the lack of ecological abundance data limits the interpretation of RFC as a proxy for species availability. Additionally, the selected indices may have influenced the results since RFC does not reflect the intensity of use and ICF may be influenced by sample size and category definition. Finally, including non-indigenous plants in the data analysis excluded the possibility of determining traditional knowledge related to the uses of native plants in the study area. Addressing these limitations in future studies will enhance the interpretability of the results.

## 4. Materials and Methods

### 4.1. Study Area

Baranja is a region located in northeastern Croatia (45°32′ N, 18°16′ E–45°55′ N, 18°57′ E; mean elevation: 97 m). It is part of the Croatian Danube area, bordered by the Drava River to the southwest, the Danube River to the east, the Croatian–Hungarian border to the northwest, and the city of Osijek to the south. The area belongs to the northern temperate climate zone and is influenced by a moderately warm continental climate. Springs and autumns are mild with frequent precipitation, summers are generally hot and humid, and winters are cold and foggy. The prevailing winds blow from the northwest and north [98].

Alluvial plains occupy the majority of the region [98]. The natural vegetation, oak forests, and steppes have largely disappeared [99]. Currently, higher areas are dominated by *Populus alba* L., *Populus nigra* L., and *Quercus robur* L., while the wettest parts of the floodplain are covered by a forest of *Salix* spp. [99].

The population is approximately 41,700, with an average density of 36 inhabitants per square km [100]. The area is ethnically diverse, with a primarily Croatian population alongside significant minorities of Hungarians and Serbs. There are also smaller German and Slovenian populations [98]. The population is predominantly engaged in agriculture, livestock farming, beekeeping, fruit farming, and viticulture [101]. Thanks to its rich natural and cultural–historical heritage, excellent gastronomy, and top-quality wines, Baranja is well known for its developed agritourism. An additional advantage of the region is its high connectivity to major regional, national, and international routes, making it easily accessible to tourists and visitors [102].

### 4.2. Data Collection

**Figure 2:**
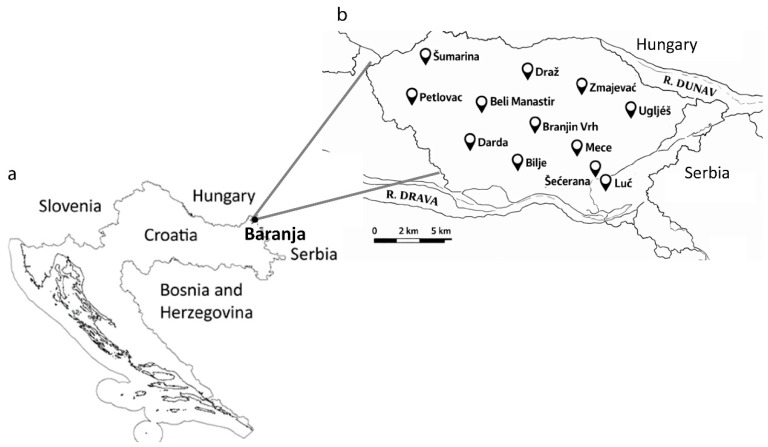
The geographical position of the Baranja region in northeast Croatia (**a**) and study sites within the region (**b**).

The field study was carried out from 2019 to 2023. Ethnobotanical data were collected through semi-structured interviews. The study was conducted in accordance with the International Society of Ethnobiology’s Code of Ethics (The ISE Code of Ethics) [103]. Informants who had significant knowledge about medicinal plants and mushrooms and their uses were selected for this study. Two co-authors from the research area, who know the local population, encouraged the informants to participate in the study. Subsequently, the snowball sampling method was applied to find additional informants. Before participating in the study, each participant was given a detailed explanation of the study’s purpose and then asked to provide verbal consent. It was additionally emphasized to the informants that all collected data will be used exclusively for the purposes of the study without specifying their names and surnames or any other data related to them. All the participants voluntarily agreed to participate in the research. A total of 105 informants from 12 settlements in Baranja (Šumarina (13), Šećerana (7), Luč (2), Darda (25), Bilje (13), Zmajevac (6), Mece (6), Petlovac (4), Beli Manastir (19), Draž (4), Branjin Vrh (4), and Uglješ (2)) participated in the interviews (Figure 2).

The majority of the informants were women (64.77%). The youngest respondent was 35 years old, the oldest was 92, and the majority of the informants were aged 46–55 (28.57%). Approximately 70% of the informants completed secondary education, while those who completed primary school and those with higher education were almost equally represented (Table 3).

The informants who agreed to participate in this study were first asked to provide basic socioeconomic details, including gender, age, place of residence, and education level. Following this, they were asked to provide information on the plants they use, their common names, the specific parts of the plants most commonly collected, the preparation methods, and the purposes for which they are used. Additionally, the informants were asked to list the mushrooms they utilize.

Plants were identified during interviews at the doorstep, in the surroundings of the house and garden, and during a field trip to the field and forest; these locations are where informants most commonly gather plants. Standard botanical keys were used for plant identification [104,105]. Identification of individual species was sometimes verified in the laboratory using a stereo microscope and a light microscope. The nomenclature of all plants cited by the informants was based on the Flora Croatica Database (FCD) [106].

The collected plants were dried and herbarized using standard methods and deposited in the botanical herbarium of the Department of Biology, University of Josip Juraj Strossmayer in Osijek.

### 4.3. Data Analysis

The data collected were sorted into Microsoft Excel and then quantitatively analyzed using the following ethnobotanical indices: the frequency of citation (FC), the relative frequency of citation (RFC) [107], the number of use reports (UR), the single-mentioned item index (SM) [108], and the informant consensus factor (ICF) [90].

The RFC index ranges from 0 to 1. A higher RFC value suggests a wider agreement on the usefulness of the plant and indicates the importance of each species; thus, a value of 1 demonstrates that all respondents recognize the species as useful. The UR value represents the total number of uses reported by each informant for a particular plant taxon. Usage reports are used to assess the consensus on the uses of plant species for various purposes. A higher SM value indicates a greater number of species mentioned only once within an ailment category, reflecting greater disagreement among informants. The ICF value also ranges from 0 to 1. A higher ICF value suggests that the majority of informants referenced a smaller number of plant species, while a lower value indicates a lack of consensus among informants regarding the species used for treating a specific ailment category.

The reported plant species were classified into 14 categories based on their medicinal properties and uses for various health conditions. These categories that follow the International Classification of Primary Health Care (ICPC-2), an international classification system for categorizing medical information in primary healthcare: (1) general and unspecified; (2) digestive; (3) blood, blood-forming organs, and immune system; (4) endocrine, metabolic, and nutritional; (5) psychological; (6) neurological; (7) eye; (8) ear; (9) cardiovascular; (10) respiratory; (11) skin; (12) musculoskeletal; (13) urological; and (14) female reproductive health.

## 5. Conclusions

This study highlights the richness of current knowledge regarding plants and mushrooms in the northeastern part of Croatia. The recorded 117 species and 7 mushroom species indicate that the local population still preserves a deep connection with nature and relies on plant-based resources for health, nutrition, and daily life. The plants most often used in the study area for various purposes are *S. nigra*, *C. recutita*, *T. officinale*, *T. cordata*, *J. regia*, and *U. dioica*, indicating their ecological abundance and cultural importance. The use of some non-indigenous plants suggests that current knowledge of plant uses reflects a blend of ethnobotanical heritage and modern knowledge. However, preserving this knowledge is crucial for developing sustainable approaches in natural resource management.

## Figures and Tables

**Table 1:** Informant consensus factor (ICF) of ailment category and the most frequently mentioned plants with the highest UR values.

Category	Plants with the Highest UR	Nur	Nt	ICF
A—general and unspecified	*Sambucus nigra* (5), *Taraxacum officinale* (2), *Brassica oleracea* ssp. *capitata* (2), *Tilia cordata* (2)	19	12	0.39
B—blood, blood-forming organs, and immune system	*Sambucus nigra* (30), *Urtica dioica* (13), *Chamomilla recutita* (11)	164	39	0.77
D—digestive	*Sambucus nigra* (18), *Mentha* × *piperita* (UR18), *Chamomilla recutita* (15)	227	55	0.76
F—eye	*Chamomilla recutita* (15), *Daucus carota* (6)	21	2	0.95
H—ear	*Sempervivum tectorum* (14)	14	1	1
K—cardiovascular	*Crataegus monogyna* (12), *Juglans regia* (8), *Malus pumila* (8)	124	46	0.63
L—musculoskeletal	*Brassica oleracea* ssp. *capitata* (9), *Symphytum* *officinale* (9), *Petroselinum crispum* (4), *Hypericum perforatum* (3)	40	18	0.55
N—neurological	*Lavandula angustifolia* (8), *Chamomilla recutita* (3), *Achillea millefolium* (2), *Galium verum* (2), *Helichrysum italicum* (2)	26	14	0.48
P—psychological	*Melisa officinalis* (21), *Hypericum perforatum* (8), *Chamomilla recutita* (5), *Thymus serpyllum* (5)	50	12	0.76
R—respiratory	*Sambucus nigra* (42), *Tilia cordata* (22), *Chamomilla recutita* (15)	176	29	0.84
S—skin	*Calendula officinalis* (20), *Chamomilla recutita* (18), *Hypericum perforatum* (17)	119	29	0.76
T—endocrine/metabolic and nutritional	*Taraxacum officinale* (8), *Juglans regia* (4), *Cucurbita pepo* (3)	42	19	0.56
U—urological	*Tilia cordata* (15), *Taraxacum officinale* (13), *Urtica dioica* (7)	88	29	0.68
X—female genital	*Bellis perennis* (5), *Achillea millefolium* (4), *Hypericum perforatum* (3)	19	10	0.5

Nur—number of use reports; Nt—number of taxa; UR—use report; ICF—informant consensus factor.

**Table 2:** Mushrooms with ethnobotanical uses in Baranja, northeastern Croatia.

Botanical Name	Local Name	Family	FC
*Cantharellus cibarius* Fr.	lisičarka	Cantharellaceae	1
*Lactarius deliciosus* (L. ex Fr.) S.F.Gray	obična rujnica	Russulaceae	1
*Lactarius piperatus* (L.) Pers.	paprena mliječnica	Russulaceae	1
*Laetiporus sulphureus* (Bull.) Murrill	vrbovača	Laetiporaceae	4
*Langermannia gigantea* (Batsch ex Pers.) Rostk.	golema puhara	Agaricaceae	1
*Pleurotus ostreatus* (Jacq. ex Fr.) P.Kumm. 1871	bukovača	Polyporaceae	1
*Sarcodon imbricatus* (L.) Karst.	srnjača	Hydnaceae	1

FC—frequency of citation.

**Table 3:** Demographic characteristics of informants (*n* = 105).

Demographical Characteristic		Number	%
Sex	Male	37	35.23
	Female	68	64.77
Age	35–45	11	10.48
	46–55	30	28.57
	56–65	19	18.10
	66–75	28	26.67
	> 75	17	16.19
Educational level	Primary school	16	15.24
	Secondary school	71	67.62
	University	18	17.14

The language of the interview was Croatian, and it was conducted orally with all informants through face-to-face interviews.

## Data Availability

The original contributions presented in this study are included in the article/Supplementary Material. Further inquiries can be directed to the corresponding author.

## Supplementary Materials

The following supporting information can be downloaded at: https://www.mdpi.com/article/10.3390/plants15020325/s1. Table S1. Plants used in Baranja, northeastern Croatia.
