# Supplementary material for: Current Knowledge and Utilization of Medicinal Plants and Fungi in Northeastern Croatia
Source: Plants (Basel). 2026 Jan 21;15(2):325. doi: 10.3390/plants15020325 (PMC12844958; doi:10.3390/plants15020325)
Supplement: Supplementary file 1 [file plants-15-00325-s001.zip › plants-4025888-supplementary.pdf]

Table S1. Plants used in Baranja, northeastern Croatia. For each plant, the scientific name, local name, family affiliation, the part used, the method of preparation, various uses, as well as the frequency of citation (FC), the relative frequency of citation (RFC), the number of use report (UR), status of the plant, and plant uses in Croatia and in other regions are listed.

Botanical name	Local name	Family	Part Used	Preparation	Uses	Status	FC	UR	RFC	Use in Croatia[1]	Use in other regions[1]
Achillea millefolium L. DB1*	obični stolisnik, stolisnik, jezičac	Asteraceae	flower, leaf	infusion, tincture, decoction	digestive problems (UR5), digestion stimulation (UR3), constipation (UR2), diarrhea (UR1), menstrual irregularity (UR4), neurosis (UR2), immunity (UR3), hypertension (UR3), blood pressure regulation (UR1), hemorrhoids (UR2), kidney disorders (UR2)	w	18	28	0.17	MED: [18][b], [22][b], [87][b],	NUT: [53], [96] MED: [1][c]. [15][b], [53][b], [66][b], [67][b], [88][c], [96][c]
Aesculus hippocastanum L. DB2*	divlji kostanj, divlji kesten	Hippocastanaceae	flower, fruit	tincture, compress, infusion	varicose veins (UR3), bone fracture (UR1)	w	4	4	0.04	MED: [18][c], [22[b]], [87][b]	NUT: [53] MED: [53[b]], [66[b]], [67[b]], [96][b]
Allium cepa L.	obični luk, crveni luk	Amaryllidaceae	bulb, onion peel, leaf	spice, fresh, infusion	respiratory problems (8), cold (1), cardiovascular (2), food, coloring eggs for Easter	c	9	11	0.09		NUT: [1, [53] MED: [1][c], [53][b], [88][a,b]
Allium sativum L.	bijeli luk, češnjak	Amaryllidaceae	bulb	raw, chopped, spice	immune system booster (6), cardiovascular (2), hypertension (1), cold (6), food	c	11	15	0.10		
Althaea officinalis L. DB3	bijeli sljez	Malvaceae	root	macerate, infusion	cough (3), laryngitis (1)	w	3	4	0.03	MED: [87][b]	MED: [53][b], [66][b], [67][b], [88][b]
Aloe vera L.	aloja	Asphodelaceae	leaf	cream	skin care (1)	c	1	1	0.01	MED: [87][b]	MED: [1][b]

Anethum graveolens L.	kopar	Apiaceae	herb	spice, fresh, cooking	gastrointestinal problems (2), food	c	2	2	0.02		
Apium graveolens L.*	celer	Apiaceae	leaf, root	infusion, soup, spice	urinary tract infection (1)	c	1	1	0.01		
Arctium lappa L. DB4	obični čičak	Asteraceae	root	tincture	hair loss (1)	w	1	1	0.01	MED: [18][c], [87][c]	MED: [66][c], [67][b], [88][c], [96][b]
*Armoracia rusticana** P. Gaertn., B. Mey. et Scherb.	hren	Brassicaceae	root	infusion	prevention of kidney stones (1)	c	1	1	0.01	NUT: [18] MED: [18][c]	NUT: [53]
Aronia sp.	aronija	Rosaceae	fruit	syrup, liqueur, jam	for cardiovascular health (4), food	c	4	4	0.04		
Artemisia vulgaris L. DB5*	pelin	Asteraceae	leaf	liqueur, infusion	digestive complaints (3), plant protection, insecticide	w	3	3	0.03	MED: [22][a,b], [87][a,b]	NUT: [53] MED: [53][b], [67][c]
Avena fatua L.	divlja zob	Poaceae	seed	boiled	mental exhaustion (1), food	w	1	1	0.01		
Bellis perennis L. DB6	obična tratinčica	Asteraceae	flower, herb	infusion	digestive problems (4), constipation (4), diarrhea (3), stomach pains (3), irregular menstruation (5), headache (1)	w	6	20	0.06	MED: [22][b], [87][c]	MED: [53][c], [96][c], [88][c]
Beta vulgaris var. *conditiva* L.	cikla	Amaranthaceae	root	pickled, fresh juice	anemia (1), food	c	1	1	0.01		MED: [53][c]
Betula pendula Roth DB7*	bijela breza	Betulaceae	leaf	infusion, compress	urinary tract infection (4), diuretic (1), skin disease (2)	w	5	7	0.05	MED: [18][c], [22][c]	MED: [15][b], [53][b], [66][b], [67][b], [96][b]
Brassica oleracea L. ssp. *capitata* (L.) Duchesne	kupus	Brassicaceae	leaf	fresh, fermented, compress, stewed	analgesic (2), osteoarthritis (2), joint pain (7), varicose veins (1), food	c	10	12	0.10	MED: [87][a,b]	MED: [53][b]

Brassica oleracea var. *viridis*	raštika	Brassicaceae	leaf	soup	diabetes (1), food	c	1	1	0.01		
Brassica napus L. ssp. *oleifera* (DC.) Janch. DB8	uljana repica	Brassicaceae	flower	honey	digestive problems (1), food	c	1	1	0.01		MED: [1][a,c], [88][a,b]
Calendula officinalis L. DB9	ljekoviti neven	Asteraceae	flower, leaf	ointment, infusion, macerate, cream, tincture, balm, compress	eczema (4), wounds (3), rash (1), insect bite (1), burns (3), dry skin (2), warts (2) skin care (4), treatment of muscle fatigue (1), gastrointestinal problems (4) varicose veins (3), hemorrhoids (3), immune system (2)	c	17	33	0.16	MED: [22][a,b], [87][b]	NUT: [53] MED: [53][b], [66][b], [67][a,b], [96][b]
Camelina sativa (L.) Crantz	usjevni podlanak	Brassicaceae	seed	oil	cholesterol (1)	c	1	1	0.01		
Capsella bursa-pastoris (L.) Medik. DB10	prava rusomača	Brassicaceae	herb	infusion, tincture	heavy menstrual bleeding (1), regulation of blood pressure (1), painful joints (1)	w	1	3	0.01	MED: [18][b]	MED: [67][b], [88][b], [96][b]
Capsicum anuum L.	paprika	Solanaceae	fruit	tincture, spice, fresh, cooking	muscle pain (1), source of vitamin C (2), immune system (2), food	c	5	5	0.05		MED: [1][c]
Centaurium erythraea Rafn DB11	kičica	Gentianaceae	herb	infusion	heartburn (1)	w	1	1	0.01	MED: [18][b], [87][c]	MED: [66][b], [67][a,b]
Ceratonia siliqua L.	rogač	Fabaceae	fruit	dry, cake, chopping	gastrointestinal problems (1), cardiovascular (1), food	c	1	2	0.01	MED: [87][b]	
Chamomilla recutita (L.) Rauschert DB12*	prava kamilica	Asteraceae	flower	infusion, compress, inhalation, cream, ointment, balm, decoction	eye infection (15), insomnia (3), anxiety (2), acne (1), burn, rash, insect bite (13), dermatological problems (4), aphthae in	w	53	87	0.50	MED: [18][b], [22][b], [87][b]	MED: [15][b], [53][b], [67][b], [96][b], [88][b]

					mouth (3), gastrointestinal problems (9), nervous stomach (2), stomach ache (1), immune system (11), sore throat (1), respiratory problems (2), cold (12), urinary tract infection (2), urological problems (1), pain (1), headache (2), neurological problems (1), menstrual problem (1)						
Chelidonium majus L. DB13*	rosopas	Papaveraceae	herb	fresh	treatment of corns (1)	w	1	1	0.01	MED: [18][c], [22][c]	MED: [53][c], [66][c], [67][c], [88][b]
Cichorium intybus L. DB14*	cikorija	Asteraceae	root	dry, chopping	gastrointestinal problems (1), coffee substitute, food	w	1	1	0.01	NUT: [18] MED: [18][b], [22][b], [87][b]	MED: [66][b], [67][c], [88][b], [96][b]
Citrus limon (L.) Burm. DB15*	limun	Rutaceae	fruit	fresh, juice	source of vitamin C (2), cough (1), immune system (2), cholesterol (1), food	c	5	6	0.05	MED: [87][b]	MED: [1][c]
Coriandrum sativum L.*	korijander	Apiaceae	leaf	infusion, spice	reduction of blood glucose (2), food	c	2	2	0.02		NUT: [1] MED: [1][c], [88][c]
Corylus avellana L. DB16*	obična lijeska	Corylaceae	fruit	dry, biscuits, cakes	heart health (3), source of minerals (1), source of vitamin E (1), food	w	5	5	0.05	NUT: [22]	NUT: [96] MED: [66][a,c], [88][c]
Cornus mas L. DB17	drijen	Cornaceae	fruit	tincture, jam, fresh	gastrointestinal problems (2), food	w	2	2	0.02	NUT: [22]	NUT: [53], [96] MED: [53][b], [66][b], [67][b], [88][b]
Crataegus monogyna Jackq. DB18	bijeli glog	Rosaceae	flower, fruit, leaf	infusion, jam, liqueur	strengthening the heart (1), hypertension (7), blood circulation (1), cholesterol (3), insomnia (2), joint health (1), source of mineral Mg (1), food	w	11	16	0.10	MED: [18][b], [22][b], [87][b]	NUT: [53], [96] MED: [66][b], [67][b], [53][a,b]

Cucurbita pepo L.	bundeva	Cucurbitaceae	fruit, seed	fresh, soup	cholesterol (1), source of vitamin A and E (2), prostate health (1), immune system booster (1), acne (1), urinary tract infection (1), food, livestock feed	c	6	7	0.06		MED: [1][b]
Cydonia oblonga Mill.	dunja	Rosaceae	fruit, leaf	infusion, fresh, compote, jam	constipation (1), stomach complaints (3), diarrhea (2), diabetes (2), urinary infections (1), food	c	6	9	0.06	NUT: [22] MED: [22][b]	MED: [66][b]
Daucus carota L.*	obična mrkva	Apiaceae	root	fresh juice, macerate, salad, soup	eyesight (1), eye diseases (5), better circulation (2), regulation of blood pressure (1), source of vitamin A (3), dry skin (3), food	c	11	15	0.10		MED: [67][b], [88][c]
Elymus repens (L.) Gould	puzava pirika	Poaceae	root	infusion	urinary infections (2), to improve blood count (2)	w	2	4	0.02	MED: [87[b]	
Equisetum arvense L. DB19*	preslica	Equisetaceae	herb	infusion, compress	heel spurs (1), gastrointestinal problems (1), immune system (1), anemia (2) diuretic (1), kidney and bladder inflammation (2), cardiovascular (2)	w	6	10	0.06	MED: [18][b], [87][b]	NUT: [53] MED: [53][b], [66][b], [67][b], [88][c], [96][b]
Foeniculum vulgare Mill.	komorač	Apiaceae	leaf	infusion, syrup, spice	gastrointestinal problems (1), cough (1), immune system (1), food	c	1	3	0.01	MED: [87][b]	NUT: [96], [53] MED: [1][c], [53][b], [96][b]
Ficus carica L. DB20	smokva	Moraceae	fruit	fresh, jam, cream	hypertension (1), viral warts (1), food	c	2	2	0.02	MED: [87][c]	NUT: [1], [53] MED: [1][c], [53][b]
Fragaria vesca L. DB21*	jagoda	Rosaceae	fruit	syrup, jam	cardiovascular (1), urinary disorders (1), food	c	1	2	0.01	MED: [18][b]	NUT: [53], [96]

											MED: [66][b], [67][c], [88][c]
Galium verum L. DB22	prava broćika	Rubiaceae	flower	infusion	kidney disorders (2), urinary tract problems (2), neurological problems (2)	w	2	6	0.02		NUT: [53][c] MED: [53][a], [66][b], [88][b]
Hedera helix L. DB23*	obični bršljan	Araliaceae	leaf	syrup, tincture	anti-inflammatory (1), cough (2), bronchitis (1)	w	4	4	0.04	MED: [87][c]	MED: [53][a], [66][b], [67][c], [88][b], [96][c]
Helianthus annuus L.	jednogodišnji suncokret	Asteraceae	seed	fresh, dry	source of vitamin E (1), source of selenium (1), cholesterol (2), food	c	3	4	0.03		MED: [1][c], [88][a,c]
Helichrysum italicum (Roth) G Don DB24*	sredozemno smilje	Asteraceae	flower	infusion	headache (2)	c	2	2	0.02		
Hordeum vulgare L. DB25*	ječam ozimac	Poaceae	seed	cooking	cholesterol (1), insulin levels (2), digestive problems (1), food, livestock feed	w	3	4	0.03		
Hypericum perforatum L. DB26*	gospina trava	Hypericaceae	flower, leaf	infusion, oil extract, macerate, compress	menopause (1), ovarian inflammation (2), skin complaints (6), wounds (5), skin care (3), burns (2), dermatological problems (1), hemorrhoids (3), cardiovascular (1), insomnia (4), antidepressant (2), calming down (2), for better digestion (2), gastrointestinal problems (1), musculoskeletal (3), immune system (1)	w	16	39	0.15	MED: [18][b], [22][b], [87][b]	NUT: [53] MED: [1][b], [15][b], [53][b], [66][b], [67][b], [88][b], [96][b]
Juglans regia L. DB27*	obični orah, pitomi orah	Juglandaceae	fruit	liqueur, fresh, cake, syrup, fruit with honey	better thyroid function (3), diabetes (1), anemia (3),	c	24	31	0.23	NUT: [18]	NUT: [53], [96] MED: [1][c], [53][b],

					immunity (2), gastrointestinal problems (9), prostate health (3), better brain function (1), cardiovascular (1), cholesterol (6), hypertension (1), cancer (1), food, insecticide					MED: [18][b], [87][b]	[66][b], [67][c], [88][b], [96][b]
Laurus nobilis L. DB28*	obični lovor	Lauraceae	leaf	syrup, infusion, spice, liqueur	cough (7), painful joints (2), gastrointestinal problems (4), immune system (3), diabetes (1), food	c	12	17	0.11	MED: [87][b]	NUT: [53] MED: [53][b]
Lavandula angustifolia Mill. DB29*	uskolisna lavanda	Lamiaceae	flower	infusion, repellent, dry flowers, ointment, spice	immune system (2), headache (8), digestive complaints (8), cold (1), laryngitis (1), cough (1), burn (1), rash (1), insect bite (1), food, decoration, repellents for mosquitoes and moths, space freshener	c	14	24	0.13	MED: [87][a,c]	NUT: [53] MED: [53][c]
Levisticum officinale Koch.*	ljupčac	Apiaceae	leaf	spice	urinary complaints (1), for better digestion (1), food	c	1	2	0.01		MED: [53][b]
Lilium sp.	ljiljan	Liliaceae	flower	tincture, compress	skin damage (1), wounds (1)	c	1	2	0.01		
Malus pumila Mill. DB30	jabuka	Rosaceae	fruit	fresh, vinegar, pie, jam, juice compote	gastrointestinal problems (1), cholesterol (8), immune system booster (1), anemia (1), food	c	8	11	0.08		
Malus sylvestris Mill. *	divlja jabuka	Rosaceae	fruit	vinegar	analgesic (1)	w	1	1	0.01		MED: [66][b]
Malva sylvestris L. DB31*	sljez	Malvaceae	flower	infusion	gastrointestinal problems (2)	w	2	2	0.02	MED: [18][b], [87][c]	MED: [66][c], [88][c]

Melissa officinalis L. DB32	ljekoviti matičnjak	Lamiaceae	flower, leaf	infusion, liqueur, fresh, syrup	for better digestion (1), to calm stress (6), insomnia (6), anxiety (9), cold (1), tachycardia (1), headache (1), reduction of menstrual cramps (1), food, beekeeping	c	16	26	0.15	MED: [18][b], [22][b], [87][b]	NUT: [53], [96] MED: [53][b], [66][b], [67][b], [88][b], [96][b]
Mentha sp.	menta	Lamiaceae	leaf	fresh, tincture, infusion	relief of nausea (1), muscle relaxation (1), respiratory problems (2)	c	3	4	0.03	MED: [18][b], [22][b], [87][b]	NUT: [53] MED: [53][b], [67][a,b]
Mentha longifolia L. DB33	dugolisna metvica	Lamiaceae	herb	infusion	influenza (1), cold (1)	w	2	2	0.02		MED: [1][c], [58][c], [66][c], [88][b]
Mentha x piperita L.*	paprena metvica	Lamiaceae	flower, leaf, herb	infusion, spice, syrup, balm, dry, fresh, liqueur	gastrointestinal problems (18), sinus infections (1), cold (2), respiratory problems (2), immune system (3), dermatological problems (2), detoxification (1), food, repellent	c	21	29	0.20		NUT: [96] MED: [96][b], [88][b]
Mentha spicata L.	klasasta metvica	Lamiaceae	herb	infusion	relaxation (2), weakened immune system (2), gastritis ulcer (1)	w	3	5	0.03		MED: [1][c]
Morus nigra L. DB34	crni dud	Moraceae	fruit	fresh	gastrointestinal problems (2), food	w	2	2	0.02	MED: [87][b]	NUT: [1] MED: [1][c], [67][b], [88][b]
Nigella sativa L.	crni kim	Ranunculaceae	seed	oil	digestive problems (1)	c	1	1	0.01		MED: [1][c], [67][c]
Ocimum basilicum L.	bosiljak	Lamiaceae	leaf	spice, fresh, infusion	kidney disease (2), gastrointestinal problems (9), calming down (2), cough (1), headache (1), food	c	10	15	0.10	MED: [87][c]	MED: [88][c]

Olea europea L. DB35*	maslina	Oleaceae	leaf, fruit	infusion, oil	cholesterol (1), hair loss (1), food	c	2	2	0.02	MED: [87][c]	MED: [53][c]
Origanum majorana L.*	pravi mravinac	Lamiaceae	herb	infusion	cough (1)	c	1	1	0.01	MED: [18][a,c], [87][c]	MED: [53][c], [66][a,b], [67][a,b]
Papaver rhoeas L. DB36*	mak turčinak	Papaveracea	seed	dry	insomnia (1)	w	1	1	0.01		MED: [67][c]
Papaver somniferum L.*	pitomi mak	Papaveracea	seed	cake, pasta with poppy seeds	relaxation (1), food	c	1	1	0.01		MED: [1][c]
Petasites hybridus (L.) P. Gaertn., B. Mey. et Schreb.	vodeni lopuh, repušina	Asteraceae	leaf	fresh	analgesic (1)	w	1	1	0.01		MED: [66][b], 67][c]
Petroselinum crispum (Mill.) A. W. Hill	peršin	Apiaceae	leaf, root	infusion, smoothie, fresh, soup	urinary tract infection (4), easier urination (1), rheumatism (2), bone health (2), regulation of blood pressure (4), heavy menstrual bleeding (1), food	c	11	14	0.10	MED: [87][c]	
Phaseolus vulgaris L.	grah	Fabaceae	seed	cooking	diabetes (1), food	c	1	1	0.01	MED: [22][c]	MED: [53][b]
Picea abies (L.) H. Karst. DB37	obična smreka	Pinaceae	leaf	tincture, syrup	cough (3), cold (1), source of vitamin C (1), immunity (1)	w	4	6	0.04	MED: [18][b], [22][c]	MED: [53][b], [96][b]
Pinus sylvestris L. DB38*	bor	Pinaceae	leaf	syrup, infusion	gastrointestinal problems (1), respiratory problems (2), immune system (2)	w	2	5	0.02	MED: [18][b], [87][a,b]	MED: [66][a,b], [67][c], [88][b], [96][a,b]
Plantago lanceolata L DB39*.	uskolisni trputac	Plantaginaceae	leaf	compress, infusion, tincture, fresh	skin infection (1), skin irritations (1), abscess (1), wounds (1), skin disease (1), immune system (1), respiratory problems (1), cough (1), cardiovascular	w	9	12	0.09	MED: [18][c], [22[b]], [87][b]	MED: [1][c], [53][b], [66][b], [67][b], [88][b], [96][b]

					(3), gastrointestinal problems (1)					
Plantago major L. DB40	veliki trputac	Plantaginaceae	leaf	compress, infusion, crushed, syrup, salad, fresh	skin inflammation (1), wounds (4), respiratory problems (1), cough (2), urinary tract infection (1), heavy menstrual bleeding (1), immune system (5), gastrointestinal problems (5), stomach disorder (3), cardiovascular (1), food	w	11	24	0.10	MED: [18][b], [22][b], [87][b]	MED: [53][b], [66][b], [67][b], [88][b]
Prunus armeniaca L.*	marelica	Rosaceae	fruit	liqueur, schnapps, fresh, jam, syrup	better digestion (3), heart health (1), muscle health (1), food	c	3	5	0.03		MED: [1][b]
Prunus avium L. *	trešnja	Rosaceae	petioles, fruit	infusion	urinary tract infection (1), food	c	1	1	0.01	NUT: [22]	
Prunus cerasus L.*	višnja	Rosaceae	fruit, seed	liqueur, seed bag, juice, cake, jam, syrup, compote	immune system (5), anemia (2), blood pressure (1), urinary disorder (1), cancer (1), better digestion (2), food	c	19	12	0.18		
Prunus domestica L*.	šljiva	Rosaceae	fruit	liqueur, syrup, jam, schnapps, pie, dry, fresh, cake, compote	better digestion (4), better circulation (3), food	c	11	7	0.10	NUT: [22]	NUT: [53] MED: [1][c], [53][b]
Prunus persica (L.) Batsch	breskva	Rosaceae	fruit	syrup, jam, schnaps	better digestion (1), food	c	2	2	0.02		
Prunus spinosa L.*	trnina	Rosaceae	flower, fruit	infusion, jam, liqueur	gastrointestinal problems (2), cardiovascular (1), food	w	2	3	0.02	NUT: [22] MED: [18][b], [87][b]	NUT: [96], [53] MED: [1][c], [53][b], [66][b], [96][c]
Pyrus sp.	kruška	Rosaceae	fruit	fresh, syrup, compote	gastrointestinal problems (2), food	c	2	2	0.02		NUT: [53] MED: [53][a,b]
Pyrus pyraster (L.) Burgsd.	divlja kruška	Rosaceae	leaf	infusion	immunity (1)	w	1	1	0.01		

Quercus sp. DB41*	hrast	Fagaceae	bark, fruit	infusion	gastritis (1), livestock feed	w	1	1	0.01	MED: [87][a,c]	MED: [53][c], [67][a,c], [96][c]
Ribes nigrum L.	crni ribiz	Grossulariaceae	fruit	fresh, jam	urinary tract infection (1), cardiovascular (1), food	c	2	2	0.02		MED: [67][a,b]
Ribes rubrum L.	crveni ribiz	Grossulariaceae	fruit	liqueur, fresh, jam	immune system (1), detoxification (1), food	c	1	2	0.01		NUT: [96]
Ribes uva-crispa L.	ogrozd	Grossulariaceae	fruit	syrup, jam	better digestion (1), food (1)	c	1	1	0.01	NUT: [22]	
Robinia pseudoacacia L. DB42*	bagrem	Fabaceae	flower	honey, infusion	headache (1), immune system (2), cold (5), analgesic (1), food	w	13	9	0.12	MED: [87][c]	NUT: [53] MED: [1][c], [66][b], [96][b]
Rosa canina L. DB43*	pasja ruža	Rosaceae	fruit	infusion, jam, liqueur	immunity (8), anti-obesity (1), source of vitamin C (1), cold (3), respiratory problems (1), gastrointestinal problems (2), better digestion (1), heart health (1), kidney disorder (1), cancer (1), food	w	17	20	0.16	NUT: [18], [22] MED: [18][b], [87][b]	NUT: [96], [53] MED: [53][b], [66][b], [67][b], [88][b], [96][b]
Rosa sp. DB44	ruža	Rosaceae	flower	infusion	laryngitis (1), blood purifier (1), immunity (2)	c	2	4	0.02		
Rosmarinus officinalis L. DB45*	ružmarin	Lamiaceae	herb, leaf, flower	spice, infusion, tincture, fresh, macerate	gastrointestinal problems (3), headache (1), cold (1), diuretic (1), hair growth (3), dermatological problems (1), immune system (5), circulation (3), rheumatism (1), mental strain and fatigue (1), food	c	18	20	0.17	MED: [87][b]	NUT: [53] MED: [53][b], [96][b]
Rubus caesius L. DB46	modrosiva kupina	Rosaceae	fruit, leaf	wine, juice, infusion, fresh, jam, liqueur	better blood count (1), anemia (3), immunity (3), better digestion (1),	w	6	9	0.06	MED: [22][b], [87][a,c]	NUT: [53] MED: [53][a,b], [66][c]

					neurological problems (1), food						
Rubus idaeus L.	malina	Rosaceae	fruit	syrup, jam, liqueur, fresh, cake	cardiovascular (2), food	c	2	2	0.02	NUT: [22]	NUT: [96]
Rumex sp.	štavelj	Polygonaceae	flower	infusion	gastrointestinal problems (2), diarrhea (1)	w	3	3	0.03	MED: [87][a,b]	
Rumex acetosa L. DB47*	kiselica	Polygonaceae	leaf	infusion	stomach disorder (1), cholesterol (1)	w	2	2	0.02	NUT: [22], MED: [18][c]	MED: [66][b], [67][a,b]
Salix alba L. DB48*	bijela vrba	Salicaceae	flower, leaf	fresh, infusion	dandruff (1), beekeeping	w	1	1	0.01		MED: [66][c], [67][c], [96][b]
Salix caprea L. DB49*	vrba iva	Salicaceae	bark, flower	compress, fresh	fever (1), decoration for the Palm Sunday holiday	w	1	1	0.01		MED: [67][a,b]
Salvia officinalis L. DB50*	kadulja	Lamiaceae	leaf, flower	infusion, syrup, compress, decoction, spice	gastrointestinal problems (8), inflammation of the gums (1), oral health (3), respiratory problems (5), sore throat (2), inhalation (1), laryngitis (3), cold (1), cough (2), immune system (9), skin infections (1), rash (2), wound (2), dermatological problems (2), bladder inflammation (2), for urinary tract (1), food, beekeeping	c	20	45	0.19	MED: [18][b], [22][b], [87][b]	NUT: [53] MED: [53][b], [67][b] [96][b]
Sambucus nigra L. DB51*	crna bazga	Viburnaceae	flower, fruit	juice, infusion, jam, syrup, elderflower fritters, liqueur	immune system (15), blood strengthening (15), cold (21), expectoration (15), flu (5), respiratory problems (1), fever (1), cancer (4), detox (2), headache (1), hemorrhoids (1), cholesterol (1), calming	w	62	103	0.59	MED: [18][b], [87][b]	NUT: [96], [53] MED: [15][b], [53][b], [66][b], [67][b], [88][b], [96][b]

					down (2), gastrointestinal problems (18), dermatological problems (1), food						
Sempervivum tectorum L.	čuvarkuća	Crassulaceae	leaf	fresh juice, infusion	digestive problems (2), immune system (1), earaches (14), corns (2), wound healing (1), heavy menstrual bleeding (1)	c	17	21	0.16	MED: [18][b], [22][b], [87][b]	NUT: [96] MED: [53][b], [66][b], [67][b], [96][b]
Silybum marianum (L.) Gaertn.*	sikavica	Asteraceae	seed, flower	oil, infusion	liver health (2)	c	2	2	0.02	MED: [22][b], [87][b]	
Solanum lycopersicum L.*	rajčica	Solanaceae	fruit	fresh, syrup	hypertension (2), food	c	2	2	0.02		
Solanum tuberosum L. *	krumpir	Solanaceae	tuber	fresh, cooking	warts (1), food	c	1	1	0.01		MED: [53][c]
Spinacia oleracea L.	špinat	Amaranthaceae	leaf	cooking, syrup	anemia (1), food	c	1	1	0.01		
Symphytum officinale L. DB52*	gavez	Boraginaceae	leaf, root, flower	fresh, compress, ointment, cream	joint problems (2), back pain (2), musculoskeletal (4), sore muscles (1), dermatological problems (2), wound healing (3), skin care (1)	w	14	15	0.13	MED: [18][b], [22][b]	MED: [53][b], [66][b], [67][b], [96][b]
Taraxacum officinale F. H. Wigg. DB53	ljekoviti maslačak	Asteraceae	flower, leaf, root	infusion, syrup, decoction, salad, honey, fresh, balm	cough (9), bronchitis (3), gastrointestinal problems (10), better digestion (3), blood purification (6), cardiovascular (2), liver health (6), blood sugar (1), diabetes (1), detoxification (8), diuretic (1), urinary tract infection (4), immune system (6), dermatological	w	43	65	0.41	NUT: [18] MED: [18][b], [22][a,b], [87][a,b]	NUT: [96], [53] MED: [1][b], [66][b], [53][b], [67][b], [88][b], [96][b]

					problems (2), osteoarthritis (1), cancer (2), food						
Thymus pulegioides L.	obični timijan	Lamiaceae	flower, herb	infusion	gastrointestinal problems (2), immune system (2), cold (1)	c	3	5	0.03	MED: [87][a,b]	MED: [67][b]
Thymus serpyllum L*.	babin timijan	Lamiaceae	herb, leaf	spice, infusion, fresh	digestive problems (1), relaxation (3), insomnia (2), food	c	4	6	0.04	NUT: [22] MED: [18][b], [22][c]	NUT: [96], [53] MED: [53][c], [66][b], [67][b], [88][c], [96][c]
Tilia sp. DB54	lipa	Malvaceae	flower	infusion	blood pressure regulation (2), rheumatism (1), calming down (1)	w	4	4	0.04	MED: [22][b]	MED: [53][b], [66][b], [96][b]
Tilia cordata Mill. DB55	sitnolisna lipa	Malvaceae	flower	infusion, honey	gastrointestinal problems (4), immune system (4), cold (20), cough (2), detoxification (15), fever (2), headache (1), food	w	26	48	0.25	MED: [18][a,b], [87][b]	MED: [53][b], [67][b], [88][b]
Trifolium pratense L. DB56	crvena djetelina	Fabaceae	flower	infusion	cholesterol (1), anti-inflammatory (1)	w	1	2	0.01		NUT: [53] MED: [53][a,c], [66][c], [88][a,c]
Urtica dioica L. DB57*	obična kopriva	Urticaceae	leaf	infusion, fresh, tincture, soup, plant protection, plant fertilizer, cooking, decoction	anemia (3), immune system (10), hair loss (3), dermatological problems (3), rheumatism (1), detoxification (4), urinary tract problems (3), gastrointestinal problems (9), respiratory problems (2), heavy menstrual bleeding (1), cardiovascular (1), cancer (1), diabetes (1), food,	w	22	42	0.21	MED: [18][b], [87][b]	NUT: [96], [53] MED: [15][b], [53][b], [66][b], [67][b], [88][b], [96][b]

					livestock feed, plant fertilizer, insecticide						
Vaccinium vitis-idaea L.*	brusnica	Ericaceae	fruit	infusion	urinary tract infection (2)	c	2	2	0.02		NUT: [96], [53][a] MED: [53][a,c], [66][b], [67][b], [88][a,c]
Valeriana officinalis L.	odoljen	Caprifoliaceae	herb	infusion	gastrointestinal problems (1)	w	1	1	0.01		NUT: [53] MED: [66][c], [67][c], [96][c]
Viola odorata L. DB58	mirisava ljubica	Violaceae	flower	decoction	face washing (1)	w	1	1	0.01	MED: [18][b]	NUT: [53][a] MED: [53][a,c], [66][c], [67][c]
Viscum album L. DB59*	bijela imela	Santalaceae	leaf	infusion	cardiovascular (2), cholesterol (1)	w	2	3	0.02		MED: [53][c], [67][c]
Vitis vinifera L. DB60	vinova loza	Vitaceae	fruit	fresh, wine, syrup	hypotension (1), anemia (3), food	c	4	4	0.04	MED: [87][c]	MED: [1][c], [53][c]
Zea mays L.	kukuruz	Poaceae	fruit	boiled corn	hypertension (4), cholesterol (1), urinary tract infection (5), food	c	6	10	0.06		NUT: [1] MED: [1][c], [53][b]

Legend: Plant part used: NUT – plant taxa used as food or plants with nutritional values; MED – plant taxa used for medicinal purposes. Status: w-wild, c-cultivated, DB-herbarium specimen number of the Department of Biology, University of Osijek, specimens were not available for all species. An asterisk (*) indicates potentially toxic effects on humans and animals, as stated according to the Flora Croatica Database (FCD) [106].

[1] Number refers to the following references: [1] Ishtiaq et al. 2024; [15] Petelka et al. 2022; [18] Žuna Pfeiffer et al. 2019; [22] Vitasović-Kosić et al. 2022; [53] Lumpert and Kreft 2017; [66] Janačković et al. 2019; [67] Redžić 2007; [87] Łuczaj et al. 2021; [88] Rexhepi et al. 2013; [96] Vitasović-Kosić et al. 2020. Symbols indicate comparison as follows: ([a]) uses of different plant species from the same genus, ([b]) similar or identical plant uses, ([c]) different plant uses.
